# The Four types of Tregs in malignant lymphomas

**DOI:** 10.1186/1756-8722-4-50

**Published:** 2011-12-09

**Authors:** Jing Wang, Xiao-Yan Ke

**Affiliations:** 1 Department of Hematology and Lymphoma Research Center, Peking University, Third Hospital, (Huayuan North Road 49#), Beijing (100191), PR China

**Keywords:** lymphomas, regulator T cells, prognosis

## Abstract

Regulatory T cells (Tregs) are a specialized subpopulation of CD4^+ ^T cells, which act to suppress the activation of other immune cells. Tregs represent important modulators for the interaction between lymphomas and host microenvironment. Lymphomas are a group of serious and frequently fatal malignant diseases of lymphocytes. Recent studies revealed that some lymphoma T cells might adopt a Treg profile. Assessment of Treg phenotypes and genotypes in patients may offer prediction of outcome in many types of lymphomas including diffuse large B-cell lymphoma, follicular lymphoma, cutaneous T cell lymphoma, and Hodgkin's lymphoma. Based on characterized roles of Tregs in lymphomas, we can categorize the various roles into four groups: (a) suppressor Tregs; (b) malignant Tregs; (c) direct tumor-killing Tregs; and (d) incompetent Tregs. The classification into four groups is significant in predicting prognosis and designing Tregs-based immunotherapies for treating lymphomas. In patients with lymphomas where Tregs serve either as suppressor Tregs or malignant Tregs, anti-tumor cytotoxicity is suppressed thus decreased numbers of Tregs are associated with a good prognosis. In contrast, in patients with lymphomas where Tregs serve as tumor-killing Tregs and incompetent Tregs, anti-tumor cytotoxicity is enhanced or anti-autoimmune Tregs activities are weakened thus increased numbers of Tregs are associated with a good prognosis and reduced numbers of Tregs are associated with a poor prognosis. However, the mechanisms underlying the various roles of Tregs in patients with lymphomas remain unknown. Therefore, further research is needed in this regard as well as the utility of Tregs as prognostic factors and therapy strategies in different lymphomas.

## Introduction

CD4^+^CD25^high^FOXP3+ Regulatory T cells (Tregs) are a specialized subpopulation of CD4^+ ^T cells, which act to suppress the activation of other immune cells, maintains the immune system homeostasis, and suppresses effector T cells in the periphery and control excessive response to foreign antigens [1]. Several Treg subsets have been identified and extensively studied. Naturally occurring Tregs represent approximately 5% to 10% of peripheral CD4^+ ^T cells in both mice and humans [2-4]. Besides naturally occurring Tregs, there are also adaptively induced antigen-specific Tregs. These cells exist in markedly higher proportions within tumor-infiltrating lymphocytes, peripheral blood lymphocytes, and/or regional lymph node lymphocytes. It has been widely acknowledged that tumor-related immunosuppression plays an important role in determining both the severity of disease and the responsiveness to therapy [5-7]. Tumor-specific Tregs require ligand-specific activation and cell-to-cell contact to exert their suppressive activity on tumor-specific effector T cells, which includes decreased cytotoxity, proliferation, and type 1 T helper cell (Th1) cytokine secretion. Through variable mechanisms, Tregs inhibit many adaptive and innate immune cells, including CD4^+ ^T cells, CD8^+ ^T cells, dendritic cells, macrophages, and B cells [8]. In recent years, it has been shown that Tregs also inhibit NK cells in a transforming growth factor-β (TGF-β)-dependent manner [9-11]. In some tumors, high numbers of Tregs are associated with a poor prognosis due to the fact that the presence of Tregs in the tumor microenvironment diminishes anti-tumor immune responses. These results might explain why current clinical trials using cancer peptides or dendritic cells (DCs) pulsed with antigenic peptides induce only transient immune responses and fail to produce therapeutic benefits [12-14].

**Figure 1:**
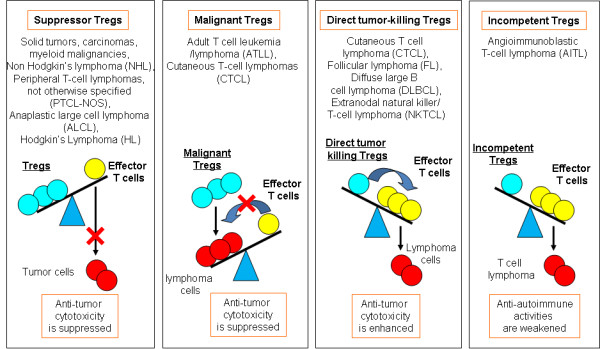
**Figure Four types of Tregs' functions have been identified in patients with lymphomas**.

Tregs represent important modulators for the interaction between lymphomas and the host microenvironment. Depletion or blockade of Tregs can enhance immune protection responses elicited by tumor-associated self-antigens. Lymphomas are a group of serious and frequently fatal malignant diseases of lymphocytes. Lymphomas are variable in their clinical features. Despite significant progress, the molecular and cellular mechanisms underlying the clinical aspects of lymphomas are largely unknown [15]. Recent studies revealed that some lymphoma T cells might adopt a Treg profile as well [16]. Assessment of Treg phenotypes and genotypes in patients may offer prediction of outcome in many types of lymphomas including diffuse large B-cell lymphoma (DLBCL), follicular lymphoma (FL), cutaneous T cell lymphoma (CTCL), and Hodgkin's lymphoma. Recent reports have shown that the presence of increased numbers of activated intra-tumoral CD4^+ ^T cells predicts a better overall survival rate in patients with lymphoma [17]. But further research is needed to clarify the complex status of Tregs in malignant lymphomas. Based on Tregs' roles characterized in lymphomas, we can categorize various roles of Tregs into four groups: (a) Suppressor Tregs: Tregs' suppression of anti-tumor CD8^+ ^T cell-mediated immune responses is observed in various lymphomas, which is similar to the ones found in solid tumors, carcinomas, and myeloid malignancies [18]; (b) Malignant Tregs: FOXP3 is a selective marker for a subset of adult T cell leukemia/lymphoma (ATLL) and cutaneous T-cell lymphomas (CTCL) suggesting that Tregs can be malignant [19,20]; (c) Direct tumor-killing Tregs: Some B cell lymphoma cells can be target cells for Tregs suppressive cytotoxicity suggesting that Tregs are tumor cell killers [21,22]; and (d) Incompetent Tregs: a reduced infiltration of Tregs, which are mostly rTreg in angioimmunoblastic T-Cell lymphoma (AITL), potentially contributing to the autoimmune symptoms, suggesting that rTregs in patients with AITL are incompetent Tregs [23] as indicated by our new working model (Figure 1). Our new classification of Tregs into four groups is significant in providing an insight into the effects of Tregs-host microenvironment interaction on Tregs' function. In addition, our new classification also predicts prognosis of these diseases and can be very useful in designing Tregs-based immunotherapies for treating lymphomas in the future. For example, in the patients with lymphomas where Tregs serve as either suppressor Tregs or malignant Tregs, anti-tumor cytotoxicity is suppressed thus decreased numbers of Tregs are associated with a good prognosis. In contrast, patients with lymphomas where Tregs serve as tumor-killing Tregs and incompetent Tregs, anti-tumor cytotoxicity is enhanced or anti-autoimmune Tregs activities are weakened thus increased numbers of Tregs are associated with a good prognosis and reduced numbers of Tregs are associated with a poor prognosis.

## Non-Hodgkin's Lymphoma

Non-Hodgkin's lymphoma (NHL) is a diverse group of lymphoid malignancies, which currently remains incurable. NHL accounts for about 90% of lymphoid neoplasms. Accumulating evidence shows that Tregs are highly concentrated in tumors, thereby fostering an immune-privileged microenvironment. Thus, newly diagnosed NHL patients are in an immunosuppressive state. Peripheral blood Tregs levels are irrelevant to the status of disease [24]. Patients with unhealthy habits (smoking, alcohol abuse) have higher peripheral blood Tregs levels. CD4^+^CD25^+ ^FOXP3^+^CD127^low ^Tregs are markedly increased in peripheral blood mononuclear cells (PBMCs) versus healthy controls regardless of lymphoma subtype, and correlated with disease stage and serum lactate dehydrogenase. A high proportion of Tregs are also present in involved tissues versus reactive nodes. Thus, NHL cells are powerful inducers of Tregs, which may represent a new therapeutic target [25]. FOXP3^+ ^Treg density is varied in different lymphoma types and is highest in follicular lymphoma.

Lymphoma-infiltrating FOXP3^+ ^cells may represent important lymphoma/host microenvironment-modulators, since increased number of these cells can positively influence survival in FL, germinal center (GC)-like DLCBL. In a Cox regression model, considering clinical stages and age, the numbers of FOXP3+ Tregs are of independent prognostic significance for disease-specific survival in GC-like DLBCL. In contrast, Tregs have a negative prognostic effect in non-GC-like DLBCL. The reason for these discrepancies remains unclear, although the expression of FOXP3^+ ^by activated Th1 cells may provide an explanation [19]. Lee *et al *indicated that an increased percentage of FOXP3-positive Tregs in DLBCL is predictive of better prognoses. As compared with the others, patients with higher percentages of FOXP3-positive Tregs on initial tumor biopsy have a significantly longer OS (*p *= 0.003). When the prognostic factors are evaluated via a multivariate model, the international prognostic index and the percentages of infiltrating FOXP3-positive Tregs in the initial biopsy are identified as independent predictors of OS [20].

Yang *et al *[26] demonstrated in NHL Tregs attenuated CD8 T-cell function, thereby protecting lymphoma cells from cytotoxic activity. Likewise, Hilchey *et al *[27] demonstrated that follicular lymphoma intratumoral Tregs suppressed CD3/CD28-costimulated autologous and allogenic CD8^+^CD25^- ^and CD4^+^CD25^- ^effector T cells. Tregs and mast cells (MCs) are involved in immunosuppression in B-cell NHL and IL-9 is a key mediator of Tregs and MCs [28]. These might provide novel insight for the pathogenesis and possible therapeutic strategy of B-cell NHL. T-cell hyporesponsiveness is reversed by depleting CD25^+ ^cells or by adding anti-CTLA-4, supporting the view that Tregs explain the systemic immunosuppression seen in NHL. Interestingly, a high level of soluble CD25 (sCD25) has been associated with a poor prognosis in patients with NHL. The function and origin of this soluble receptor are not well investigated. Tregs may release CD25 to act as a decoy receptor for IL-2, thereby depriving T-effector cells of growth factor IL-2 [29]. Thus, in patients of different types of NHL, the percentage and the function of tumor-infiltrating FOXP3+ T cells predicts different survival. Further research is needed in the utility of Tregs as prognostic factors and therapy strategies.

## Follicular Lymphoma

Follicular lymphoma (FL) is the second most common subtype of adult B-cell NHL in Western countries and is characterized by the chromosome translocation t (14;18). This abnormality promotes tumor cell survival through overexpression of the anti-apoptotic protein Bcl-2. Of interest, mice overexpressing Bcl-2 under the control of the immunoglobulin enhancer develop follicular hyperplasia rather than FL, indicating that pathogenetic mechanisms other than Bcl-2 over expression exist in FL [30]. In contrast to other B-cell lymphomas where gene expression in the malignant B-cell population determines survival, the composition of the lymphoma microenvironment at diagnosis seems to determine the survival of patients with FL [31]. Some studies have shown that stimulation of the T cell antigen receptor (TCR) or priming with dendritic cells can induce FOXP3 expression and the acquisition of Tregs activity in CD4^+^CD25^- ^T cells from normal PBMC [32-34]. Yang *et al *[35] extended these findings and demonstrated that tumor infiltrating T cells in FL-involved lymph nodes could also be induced to express FOXP3 through TCR stimulation. Furthermore, they showed that CD70^+ ^malignant B cells could facilitate this conversion of conventional T cells to Tregs in FL. Ai *et al *found that tumor B cells alone, without artificial TCR stimulation, could induce conventional T cells to express FOXP3 and acquire regulatory function. In contrast to their malignant counterpart, normal B cells did not induce Tregs conversion. Tregs conversion is independent of the T cell background, as T cells isolated from FL or normal peripheral blood are equally susceptible to being converted by tumor B cells. There is a tumor-specific mechanism by which FL tumor cells promote immune escape through the induction of Tregs [36].

In a study where Tregs are detected in FL patients, the median Tregs percentage at diagnosis is 10.5%. Overall, 49 patients have more than 10% Tregs, 30 have between 5% to 10%, and 19 have less than 5%, with a 5-year OS of 80%, 74%, and 50%, respectively (*p *= 0.001). Patients with very low numbers of Tregs (< 5%) are associated with high frequencies for refractory disease (*p *= 0.007). The prognostic significance of Tregs numbers is independent of the FL International Prognostic Index (FLIPI). Seven transformed diffuse large B-cell lymphomas (DLBCLs) have lower Tregs percentages (mean: 3.3%) than FL grades 1,2 (mean: 12.1%) or 3 (mean: 9%) (*p *< 0.02). These results demonstrate that the number of tumor-infiltrating FOXP3-positive Tregs is a predictor of survival in patients with FL; high Treg numbers predict improved survival and the number of these cells decreases during the transformation to DLBCL. Tregs may be an important subset of cells in the tumor microenvironment modulating the host immune response and biologic behavior of FL [37]. In conclusion, FL tumor cells can promote immune escape through the induction of Tregs, whereas Tregs also can serve as direct tumor-killing Tregs in FL.

## Adult T-cell leukemia/lymphoma

Adult T-cell leukemia/lymphoma (ATLL) is an aggressive lymphoma associated with human T-lymphotrophic virus 1 (HTLV1) infection, and ATLL cells frequently express several molecules that are characteristic of Tregs, notably CD4, CD25, and the transcription factor FOXP3. However, this phenotype is not characteristically found in other lymphomas [38]. Therefore it has been recently suggested that HTLV-1 selectively infects and transforms Tregs [39]. It is expected that HTLV-1 infected cells should be recognized and eradicated by HTLV-1-specific cytotoxic T lymphocytes (CTLs). However, HTLV-1 specific CTLs are suppressed by Treg activities. The reason is that HTLV-1 infected Tregs may have a survival advantage compared to other types of cells by suppressing host immune responses against themselves. As a result, HTLV-1 infected CD4^+^CD25^+^CCR4^+ ^Tregs should preferentially survive, and gradually increase in numbers. Furthermore, the accumulation of additional crucial genomic and ⁄ or epigenomic alterations could cause HTLV-1 infected cells to change into clonally proliferating ATLL cells.

Bangham *et al *showed that HTLV-1 induces and maintains a high frequency of FOXP3^+ ^T cells by inducing expression of the chemokine CCL22; the frequency is especially high in patients with chronic ATLL. In turn, the FOXP3^+ ^T cells exert both potentially beneficial and harmful effects: they may suppress the growth of autologous ATLL clones and also may suppress the host's cytotoxic T lymphocyte response, which normally limits HTLV-1 replication and reduces the risk of HTLV-1 infection-associated diseases. Although ATLL cells may exert immune suppressive effects, ATLL is not necessarily a tumor of classical FOXP3^+ ^Tregs [40]. Thus, Tregs in ATLL partly serve as malignant Tregs and lead to progress of the disease.

## Cutaneous T-Cell Lymphoma

Cutaneous T-cell lymphomas (CTCLs) are non-Hodgkin's lymphomas derived from T cells that home to and inhabit the normal function and structure of skin. There are conflicting reports as to whether CTCLs represent a malignancy of Tregs. Sézary syndrome (SS) is an aggressive variant of cutaneous T-cell lymphoma. Heid *et al. *presented convincing evidence that the malignant T cells in a subgroup of Sézary patients are FOXP3^+ ^Tregs. Clonal malignant T cells show increased expression of the Treg-associated transcription factor FOXP3 and demethylation of the foxp3 gene locus, and T cells from some of these patients suppressed T-cell proliferation *in vitro *[41]. Mycosis fungoides (MF) with early or infiltrated plaques have significantly higher numbers of FOXP3+ Tregs than CTCL unspecified or advanced MF with tumors or transformation to large cell lymphoma. An analysis of CTCL patients demonstrated that increased numbers of FOXP3+ Tregs are associated with improved survival in both MF and CTCL unspecified [42]. Knol *et al. *showed that functional circulating CD4^+^CD25^high ^FOXP3^+ ^regulatory T-cells in CTCL patients are increased compared to that in healthy donors [43]. FOXP3-positive T cells occur in higher proportions in the dermis than in the epidermis and probably correlate with coexisting inflammatory components [44].

In addition to that discussed above, others have contrary opinions. Some studies showed that CTCL neoplastic cells do not typically express a Tregs phenotype and are associated with low numbers of FOXP3-positive Tregs in the infiltrate. FOXP3-positive T cells are less frequently encountered in MF than in inflammatory dermatoses. In eight of 15 (53%) Sézary patients, significantly reduced percentages of FOXP3^+ ^cells are seen in blood (2.9%) and skin (10.4%). Interestingly, six of 15 (40%) Sézary patients show significantly increased percentages of FOXP3^+ ^cells (39.7% (blood), 20.3% (skin)); however, these cells do not express CD25. In these latter patients, clone-specific TCR-Vβ-chain antibodies were used to demonstrate that these FOXP3^+^CD25^- ^cells are monoclonal CTCL tumor cells. FOXP3+CD25- CTCL tumor cells show a highly demethylated status of the Foxp3 gene locus similar to Tregs, and they are functionally able to suppress IL-2 mRNA induction in TCR-stimulated conventional T cells. Thus, FOXP3^+^CD25^- ^CTCL tumor cells with functional features of Tregs define a subgroup of Sézary patients who might carry a different prognosis and might require different treatments [45].

In an extracorporeal photopheresis (ECP) treatment group, the baseline of circulating CD4^+^CD25^+bright ^percentages in CTCL (median: 4.3 percent) are similar to those of healthy donors in a study. During ECP treatment, CTCL patients are characterized by an early decrease of CD4^+^CD25^+bright ^(from 4.3 percent to 2.4 percent median after 6 months). The CD4^+^CD25^+bright ^decrease is associated to the disease course, as it occurs in 91.3 percent of patients responding to treatment but in only 25 percent of progress disease (PD) patients (*p *= 0.0001). ECP induces downregulation of circulating CD4^+^CD25^+bright ^cells in CTCL is potentially associated with response mechanisms [46].

Tiemessen *et al. *found a dysfunction of peripheral Tregs in certain CTCL patients, which correlates with tumor burden. The percentages of Tregs do not differ significantly between patients and controls. Functional assays demonstrates a dichotomy in Tregs function: in four out of 10 patients, CD4^+^CD25^+ ^T cells are incapable of suppressing autologous CD4^+^CD25^- ^T-cell proliferation, whereas suppressive function is intact in the other six patients. Suppressive activity of Tregs inversely correlates with the peripheral blood tumor burden. Mixed lymphocyte reactions demonstrate that CD4^+^CD25^- ^T cells from patients who lack functional Tregs are susceptible to suppression by Tregs from healthy controls, and have not become suppressive cells. Furthermore, expression of FOXP3 is reduced in the CD4^+^CD25^+ ^Tregs of these patients compared to the other six CTCL patients and controls [47].

Immunodeficiency develops during Sézary syndrome (SS) progression. Krejsgaard *et al. *studied Tregs function and the expression of FOXP3 in SS. Eight of 15 patients stain positive with an anti-FOXP3 antibody in malignant T cells. Western blotting analysis shows expression of two low molecular splice forms of FOXP3, but not of wild-type (WT) FOXP3. Malignant T cells produce interleukin-10 and transforming growth factor-β (TGF-β) and suppress the growth of non-malignant T cells. The Tregs phenotype and the production of suppressive cytokines are driven by aberrant activation of Jak3 independent of the FOXP3 alternatively spliced forms. In contrast to WT FOXP3, the low molecular splice forms of FOXP3 have no inhibitory effect on nuclear factor-κB (NF-κB) activity in reporter assays, which is in keeping with a constitutive NF-κB activity in malignant T cells. Thus, malignant T cells express low molecular splice forms of FOXP3 and function as Tregs. Furthermore, FOXP3 splice forms are functionally different from WT FOXP3 and are not involved in the execution of the suppressive function [48]. In conclusion, the percentage and function of tumor-infiltrating FOXP3^+ ^T cells predicts different survival. Tregs in CTCLs serve as both malignant Tregs and direct tumor-killing Tregs.

## Extranodal natural killer/T-cell lymphoma

Extranodal natural killer/T-cell lymphoma (NKTCL) usually derives from natural killer (NK) cells or, rarely, from cytotoxic T cells. NKTCL is common in East Asians and Mexicans, but rare in Western populations [49]. NKTCL is often refractory to radio- or chemotherapy and shows aggressive behaviors with poor clinical outcomes. Therefore, risk stratification of NKTCL is important for the appropriate management of affected patients. However, the role of alleged risk factors of non-Hodgkin's lymphoma creates limitations in evaluating the prognosis of NKTCL patients. Clinical heterogeneity and the controversial issues on the prognostic factors impose requirements for a new approach to dealing with NKTCL.

Studies show that the percentage of FOXP3^+ ^subset from PBMCs in extranodal NK/T cell lymphoma (ENKTL) patients is significantly higher than that of healthy individuals (*p *< 0.001). The FOXP3^+ ^subset from PBMCs expressed CD45RO, CTLA4, GITR, CCR7, and had an IL-10^high ^IFN-γ+ TGF-β+ IL-2^low ^IL-17^low ^cytokine secreting phenotype. Interestingly, the existence of EBV antigen-specific CD8^+ ^FOXP3^+ ^Tregs is discovered in ENKTL. Furthermore, the high density of FOXP3^+ ^tumor infiltrated lymphocytes (TILs) is associated with improved progression-free survival (PFS) in ENKTL patients (*p *< 0.05) [50]. Kim *et al. *also found that NK/T cell lymphoma (NKTCL) patients with increased numbers of Tregs (> or = 50/0.40 mm^2^) show prolonged overall and progression-free survival. In their study, tumor-infiltrating FOXP3^+ ^Tregs are much rarer in non-upper aerodigestive tract (UAT)-NKTCLs than in UAT-NKTCLs and in patients with poor performance status (PS), which might lead to poor clinical outcome of patients with decreased Tregs. However, in multivariate analysis, a paucity of Tregs is independently associated with poor clinical outcome of NKTCL patients regardless of the primary site involved and the PS. Furthermore, the quantity of Tregs has an independent prognostic value in analyses confined to UAT- or nasal NKTCLs. Therefore, it is possible that Tregs might infiltrate the NKTCL in a different manner depending on the site involved, but still have prognostic value irrespective of primary tumor site. Moreover, the independent prognostic implication of the quantity of Tregs is consistently observed in analyses of NKTCL patients treated with homogeneous modality. These observations suggest that infiltration of FOXP3^+ ^Tregs in the tumor microenvironment might reflect a unique and clinically important biological aspect of NKTCL [51]. In NKTCL, Tregs might serve as direct tumor-killing Tregs.

## Peripheral T-cell Lymphoma, not otherwise specified

Peripheral T-cell lymphomas, not otherwise specified (PTCL-NOS), are biologically heterogeneous and have not been successfully correlated with specific T-cell subsets. Bonzheim *et al. *found that one PTCL-NOS case strongly expresses FOXP3 in the neoplastic T cells and shows unusual histomorphologic features with a dense infiltration of the lymph node by immunoblastic T cells and with almost no reactive background infiltrate. The patient died shortly after diagnosis. Although the majority of patients with PTCL-NOS have a poor clinical outcome, the rapid and fatal progression seen in the FOXP3+ case differs from the typical clinical course of PTCL-NOS in general. Thus, FOXP3-expression and the associated regulatory phenotype might be an adverse biologic and clinical factor in rare PTCL cases that contributes to the aggressiveness of the tumor. All remaining PTCL-NOS cases show FOXP3 positivity only in the reactive infiltrate. FOXP3+ PTCL-NOS presumably derived from *bona fide *Tregs occurs but is rare in the Western population [52]. Like solid tumors, carcinomas, and some type of NHL, Tregs in PTCL-NOS might serve as suppressor Tregs and the anti-tumor cytotoxicity of effector T cells is suppressed in this disease.

## Angioimmunoblastic T-cell Lymphoma

Angioimmunoblastic T-cell lymphoma (AITL) is one of the nodal T-cell lymphomas and is characterized by lymphadenopathy, B-symptoms, and an aggressive behavior. Its natural history has been the subject of controversy, having been considered for many years to be non-malignant disorders or dysimmune diseases. CD4^+^CD25^high^FOXP3^+^CD127^low ^Tregs in the tumor microenvironment play an important role in lymphoma growth regulation. Numbers of Tregs are significantly decreased in AITL lymph nodes compared with follicular lymphoma and reactive lymph nodes. Moreover, the few Tregs in lymph nodes of AITL are resting Tregs (rTregs) and have a naive CD45RA^+^, PD1^-^, and ICOS^- ^phenotype, in contrast to the Tregs in follicular lymphomas or reactive lymph nodes [53]. This phenotype may potentially contributes to the autoimmune symptoms, as described in systemic lupus [54] and could participate in the poor prognosis of AITL. Interestingly, Tregs depletion is not observed in AITL peripheral blood at diagnosis. These data suggest that Tregs depletion could contribute to the nodal neoplastic T (FH) expansion and dysimmune symptoms in AITL. However, the cause of this Tregs depletion and the mechanisms of interaction between AITL neoplastic T-cells and Tregs remain to be defined. PD1 is known to be expressed by the AITL neoplastic T-cells and is also involved in the Tregs negative regulation. It is possible that the PD1 pathway is involved in down-regulation of the Tregs population observed in AITL [55]. This result indicated that rTregs in AITL are incompetent Tregs; anti-autoimmune Tregs activities are weakened and reduced numbers of Tregs are associated with a poor prognosis.

## Anaplastic Large Cell Lymphoma

Primary cutaneous CD30+ lymphoproliferative disorders (LPDs) are the second most common group of cutaneous T-cell lymphomas (CTCLs), accounting for approximately 30% of CTCLs. This group includes primary cutaneous anaplastic large cell lymphoma (C-ALCL), lymphomatoid papulosis (LyP), and borderline cases. It is now generally accepted that C-ALCL and LyP form a spectrum of disease, and that histologic criteria alone are often insufficient to differentiate between these two ends of this spectrum. FOXP3 expression in cutaneous and systemic CD30^+ ^lymphoproliferations is generally confined to tumor infiltrating Tregs. These cells may have influence upon the clinical behavior, possibly depending upon the net degree of Tregs mediated immune suppression of tumor cells relative to tumor infiltrating, cytotoxic effector cells, thereby implicating the more favorable outcome of LyP compared to C-ALCL [56]. Only a subset of tumor cells in anaplastic lymphoma kinase (ALK)+ ALCL expresses FOXP3, and the level of expression varies among tumor cells, pointing to an intricate mechanism of FOXP3 regulation. Recent data suggest that FOXP3 may be up-regulated as a consequence of ALK deregulation in ALCL, as it has been shown in ALK+ ALCL cell lines. Kasprzycka *et al. *[57] demonstrated elevated FOXP3 expression in ALK+ ALCL cell lines. When transfected with nucleophosmin-anaplastic lymphoma kinase (NPM/ALK), the lymphoid cells can be induced to express Tregs phenotype. Inhibition of NPM/ALK function in ALK+ cell can suppress Tregs phenotype. Furthermore, NPM/ALK induced Tregs phenotype by activating its key effector, signal transducers, and activators of transcription protein 3 (STAT3) to activate interleukin-10 (IL-10), transforming growth factor β, and FOXP3 expression. These findings identified a function for NPM/ALK as an inducer of evasion of the immune response. Advanced research is needed to explore the Tregs in ALCL. Tregs may serve as the suppressor Tregs in ALCL and suppress the anti-tumor immune response induced by effector T cells.

## Hodgkin's Lymphoma

Hodgkin's lymphoma (HL) is characterized by the presence of a small number of tumor cells in a rich background of inflammatory cells, but the contribution of the abundant non-tumor cells to HL pathogenesis is poorly understood. Migratory CD4^+ ^cells induced by HL cells are hyporesponsive to T-cell receptor stimulation and suppress the activation/proliferation of the effector CD4^+ ^T cells in an autologous setting. HL cells in affected lymph nodes are surrounded by a large number of lymphocytes expressing both CC chemokine receptor 4 (CCR4) and FOXP3 [58]. The functional role of these TILs is still controversial. While generally considered to represent an anti-tumor immune response, TIL in classical HL (cHL) might result from the profoundly deregulated immunity of cHL patients.

Kelley *et al. *studied the prognostic importance of tumor-infiltrating regulatory T lymphocytes (Tregs) and cytotoxic T/NK lymphocytes (CTLs) in 98 diagnostic biopsy specimens from patients with cHL. When prior available MAL and bcl-2 expression data are included in a multivariate analysis of all clinical and biologic factors, a FOXP3/GrB ratio of 1 or less and tumor cell expression of MAL and bcl-2 all independently predicted poor failure-free survival (FFS) [59]. Tumor-infiltrating CTLs, T helper 1 (Th1) cells, T helper 2 (Th2) cells, and Tregs are detected to evaluate the prognostical significance. The results show that in cHL, the microenvironment is dominated by Th2 cells and Tregs; large numbers of Th2 cells are associated with significantly improved disease-free survival (*p *= 0.021) and event-free survival (*p *= 0.012). Furthermore, a high ratio of Tregs over Th2 cells results in a significantly shortened disease-free survival (*p *= 0.025). These observations suggest that Tregs may exert inhibitory effects on anti-tumor immune responses mediated through Th2 cells and that Th2 cells may be more important for effective anti-tumor immunity than anticipated [60]. Furthermore, an increased number of Tregs in HL is associated with the loss of EBV-specific immunity. Baumforth *et al. *found that in EBV- infected HL cell lines, the higher levels of Chemokine (C-C motif) ligand 20 (CCL20) in the supernatants increase the migration of CD4^+ ^lymphocytes that express FOXP3, a marker of Tregs. Inducing the expression of CCL20 might be a mechanism by which EBV can recruit Tregs to the microenvironment of HL and, by doing so, prevent immune responses against the virus-infected tumor population [61].

The malignant Hodgkin/Reed-Sternberg (HRS) cells of classical Hodgkin lymphoma (HL) are derived from mature B cells, but have lost a considerable part of the B cell-specific gene expression pattern. Consequences of such a lineage infidelity for lymphoma pathogenesis are currently not defined. HRS cells aberrantly express the common cytokine-receptor gamma-chain (gamma(c)) cytokine IL-21, which is usually restricted to a subset of CD4^+ ^T cells, and the corresponding IL-21 receptor. IL-21 activated STAT3 in HRS cells, up-regulates STAT3 target genes, and protects HRS cells from CD95 death receptor-induced apoptosis. Furthermore, IL-21 is involved in the up-regulation of CC chemokine macrophage-inflammatory protein-3α (MIP-3α) in HRS cells. MIP-3α in turn attracts CCR6^+^CD4^+^CD25^+^FOXP3^+^CD127^low ^regulatory T cells toward HRS cells, which might favor their immune escape. Together, these data support the concept that aberrant expression of B lineage-inappropriate genes plays an important role for the biology of HL tumor cells [62]. Thus, the Tregs in HL may serve as suppressor Tregs, and is associated with the loss of EBV-specific immunity.

## Tregs and tumor immunotherapy

Tumor-induced immunotolerance mediated by inducible Treg (iTreg) is a major obstacle to tumor immunotherapy. Tregs induced by a shared idiotype epitope can systemically suppress T cell responses against idiotype-derived and immunodominant foreign epitopes *in vivo*. So tumor vaccines should avoid epitopes expressed by normal cells in the draining lymph node to achieve optimal anti-tumor efficacy [63]. In a basic study of immunotolerance, injection of an endogenous superantigen, i.e. the minor lymphocyte stimulatory (Mls)-1(a), into specific TCR Vβ8.1-transgenic (Tg) mice enables generation of anergic CD25^- ^iTreg, the immunosuppressive function of which is maintained by IL-10 production via p38-mitogen-activated protein kinase (MAPK) activation. Ovalbumin peptide OVA (323-339) iv. injection into its specific TCR-Tg (OT-II) mice also induces adaptive tolerance by iTreg. Peptide immunotherapy with p38-inhibitor after CD25^+ ^Treg-depletion is performed in an OVA-expressing lymphoma E.G7-bearing tolerant model establishes by adoptive transfer of OT-II CD25^- ^iTreg, which results in suppression of tumor growth. Similarly, the antitumor immunity induced by peptide immunotherapy in colon carcinoma CT26-bearing mice, in which the number of IL-10-secreting iTreg is increased, is augmented by treatment with p38-inhibitor after CD25^+ ^Treg-depletion and results in inhibition of tumor progression. These results suggest that simultaneous inhibition of two distinct Treg-functions may be important to the success of tumor immunotherapy [64]. Tregs depletion in mice leads to tumor rejection that is dependent on T cells, natural killer (NK) cells, and IFN-γ. In the absence of Tregs, elevated level of IFN-γ are produced by tumor-infiltrating T cells and NK cells. Tumor rejection observed in the absence of Tregs correlates with a substantial IFN-γ-dependent increase in the numbers of tumor-infiltrating leukocytes. The most abundant cell populations in the tumors are macrophages. Tumor-infiltrating macrophages from Tregs-depleted mice express increased amounts of major histocompatibility complex (MHC) class II, produce highly enhanced levels of pro-inflammatory cytokines, and inhibit tumor cell proliferation. It has been reported that tumor-infiltrating macrophages have multi-faceted functions promoting or counteracting tumor growth. High numbers of macrophages infiltrating MHC class I-deficient tumor cell line (RMA-S) tumors in the absence of Tregs correlate with tumor rejection suggesting that macrophages are additional targets for Tregs-mediated immune suppression in cancer [65].

Since many types of lymphoma are Epstein Barr Virus (EBV) positive, there are several strategies of targeting Tregs in the immunotherapy against EBV-positive lymphoma [66]. (1) Manipulating Tregs functions by depleting Tregs or blocking its suppressive regulatory molecules may provide a novel therapeutic approach for EBV-positive lymphomas. (2) The EBV epitope antigens, which can activate a Th1 response but not a regulatory T cell type 1 (Tr1) regulatory response should be considered, and the concentration of peptides vaccine should also be carefully evaluated for the vaccination against EBV positive lymphomas. (3) Depletion of Tregs should be performed before infusion of EBV specific CTL to enhance the clinical therapeutic benefit of CD8^+ ^EBV-specific CTL immunotherapy. (4) Selection of suitable timepoints for infusing CTL into the patients is important to attenuate the inhibitory effect of Tregs on the EBV-specific CTL adoptive immunotherapy [67].

## Conclusion

The regulatory T cells - Tregs - play an important role in malignant lymphomas, but much remains to be clarified. Differed from epithelial carcinomas, Tregs in lymphoma must be more complex. In patients with different types of lymphomas, the percentage of tumor-infiltrating FOXP3^+ ^T cells predicts different survival and prognosis. Not only the percentages but also the functions of Tregs are critical. Our new classification of Tregs into four groups is significant in providing an insight into the effects of Tregs-host microenvironment interaction on Tregs' function. In addition, our new classification also predicts prognosis of the diseases and is very useful in designing Tregs-based immunotherapies for treating lymphomas in the future. Therefore, it is possible that the effect of Tregs fundamentally differs in lymphoid malignancies compared with epithelial carcinomas. Further research is needed in determining how to use Tregs as prognostic factors and therapy strategies in treating different lymphomas.

## Competing interests

The authors declare that they have no competing interests.

## Authors' contributions

JW wrote the paper, XYK designed and revised the paper. All authors read and approved the final manuscript.
